# Treatment of toxic epidermal necrolysis in a patient with extensive skin damage induced by dimethyl N-cyanodithioiminocarbonate: a case report

**DOI:** 10.3389/falgy.2026.1765772

**Published:** 2026-04-01

**Authors:** Xiaokun Tian, Kai Zhang, Yingwei Qu, Bin Xu, Kaixin Cao, Cheng Wang

**Affiliations:** Department of Burn, Plastic and Wound Repair, The First Hospital of Zibo, Zibo, Shandong, China

**Keywords:** burn care, dimethyl N-cyanodithioiminocarbonate, multidisciplinary management, occupational exposure, organic compound, toxic epidermal necrolysis

## Abstract

**Introduction:**

Dimethyl N-cyanodithioiminocarbonate is a sulfur-containing industrial compound known to induce severe cutaneous reactions, including toxic epidermal necrolysis (TEN). Current management primarily focuses on immunomodulation, yet there is a recognized gap in standardized wound and systemic care for extensive, sepsis-complicated cases. This report describes a rarely documented case of dimethyl N-cyanodithioiminocarbonate-induced TEN involving over 90% of the total body surface area (TBSA) complicated by wound sepsis, and represents the application of a major-burn treatment protocol for this specific chemical-induced condition.

**Case presentation:**

A 43-year-old male chemical plant worker presented with rapidly progressing skin detachment covering 92% TBSA and mucosal involvement, following occupational exposure without adequate protection. After initial dermatological management failed to prevent clinical deterioration, he was transferred to a burn intensive care unit (BICU). A multidisciplinary burn-oriented strategy was implemented, including: precise fluid resuscitation, gradual tapering of systemic corticosteroids, use of an air-fluidized bed, topical nano-silver dressings with compound purple grass oil, targeted antibiotics, and intensive nutritional support. Within two weeks, complete re-epithelialization was achieved without surgical intervention, and all infection markers normalized.

**Conclusions:**

The integration of burn-critical care principles—particularly structured fluid resuscitation, pressure-offloading, and moist wound management—into the treatment of severe chemical-induced TEN can facilitate rapid wound closure and systemic recovery even in high-mortality-risk cases. This case supports the adoption of a multidisciplinary, burn-informed approach for extensive TEN and underscores the need for heightened occupational safety measures when handling potent sensitizers such as dimethyl N-cyanodithioiminocarbonate.

## 1 Introduction

Dimethyl N-cyanodithioiminocarbonate (CAS 10191-60-3) is a sulfur-containing organic compound widely used in the synthesis of pharmaceuticals, pesticides, and perfumes (1). Occupational or accidental exposure to this chemical can induce severe cutaneous adverse reactions, most notably toxic epidermal necrolysis (TEN), a life-threatening condition characterized by extensive epidermal detachment and mucosal involvement. TEN shares remarkable pathophysiological similarities with major burns, including profound skin barrier disruption, substantial fluid and protein loss, and high susceptibility to infection and sepsis. Despite these parallels, current management strategies for chemically induced TEN—particularly in cases triggered by industrial sensitizers such as dimethyl N-cyanodithioiminocarbonate—remain largely anchored in dermatological and immunosuppressive approaches, often overlooking the systemic and wound-specific challenges that resemble those of major burns (2).

Traditional TEN care emphasizes immunomodulation with corticosteroids or intravenous immunoglobulin, alongside supportive measures (3). However, in patients with extensive skin involvement exceeding 90% of the total body surface area (TBSA) and complicated by wound sepsis, such regimens frequently prove insufficient in preventing hypovolemic shock, organ dysfunction, and infective complications (4). There is a notable gap in clinical guidelines regarding the integration of burn-specific resuscitation, wound care, and multidisciplinary intensive care into the management of severe chemical-induced TEN—a therapeutic void that may contribute to delayed recovery and elevated mortality in high-risk cases.

Here we report a rarely documented case of dimethyl N-cyanodithioiminocarbonate-induced TEN involving 92% TBSA and complicated by wound sepsis, for which a comprehensive major-burn treatment protocol was successfully implemented. This case highlights the potential utility of adopting burn-critical care principles—including precise fluid resuscitation, air-fluidized bed support, advanced wound dressings, and structured infection control—in the setting of severe chemical-induced epidermal necrolysis. By bridging burn care and dermatological management, this approach offers a clinically reasoned strategy to address the systemic and local challenges of extensive TEN, with implications for therapeutic standardization and outcome improvement in similarly affected patients.

## 2 Case description

The patient was a 43-year-old male chemical plant worker with no chronic medical conditions, such as diabetes mellitus, hypertension, or autoimmune diseases. He had no history of allergies to drugs, chemicals, or food, and no family history of severe cutaneous adverse reactions (e.g., toxic epidermal necrolysis, Stevens-Johnson syndrome) or autoimmune disorders. Prior to this admission, he had no history of mental health issues or substance abuse, and he was fully independent in his daily living activities.

He presented with localized erythema and pruritus on the hands and forearms approximately 3 days after occupational exposure to the white powder-like dimethyl N-cyanodithioiminocarbonate via dermal contact due to not wearing occupational protective gloves. Despite effective rinsing administered within a few minutes after contact, over the next week, the skin lesions rapidly progressed to involve the trunk, face, neck, and perineum, accompanied by painful blistering and widespread epidermal detachment. He was initially admitted to a local dermatology department, where immunotherapy and anti-infection treatment were administered on the 7th after the onset of the disease. Initial treatment included intravenous methylprednisolone (80 mg daily), human immunoglobulin (0.4 g/kg/day for 3 days), and broad-spectrum antibiotics.

**Figure 1:**
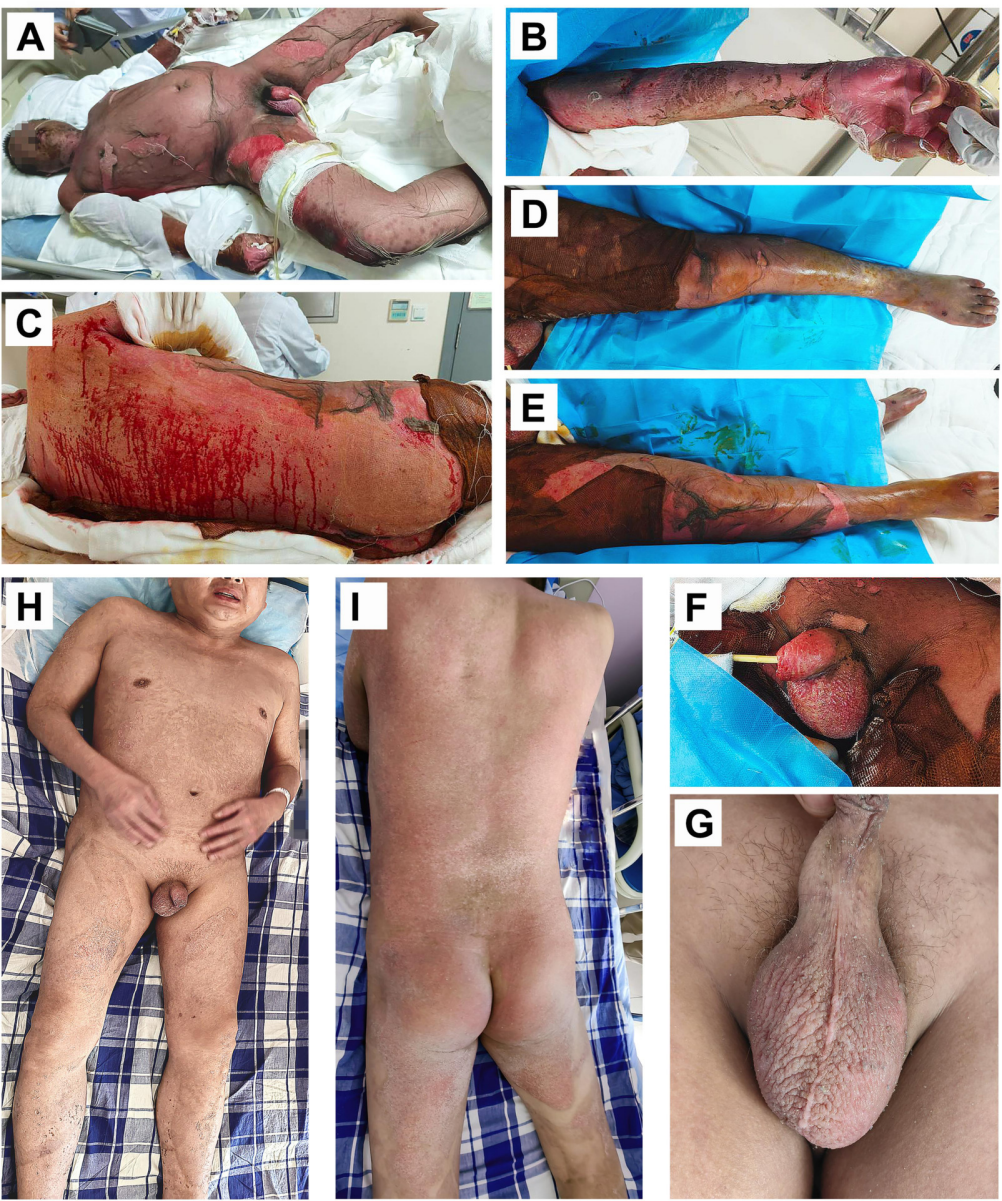
Representative images depicting skin injury with extensive peeling and exposed dermis on the 24th day (21st October) post-onset, and after two weeks of treatment, the skin wound has healed. **(A)** Whole-body skin lesions affect 92% of the total body surface area (TBSA); **(B–E)** extensive skin sloughing affecting the upper limb and trunk; **(F)** painful mucous membrane damage in the genital region; **(G–I)** whole body skin wound healing, including genital mucosa.

**Figure 2:**
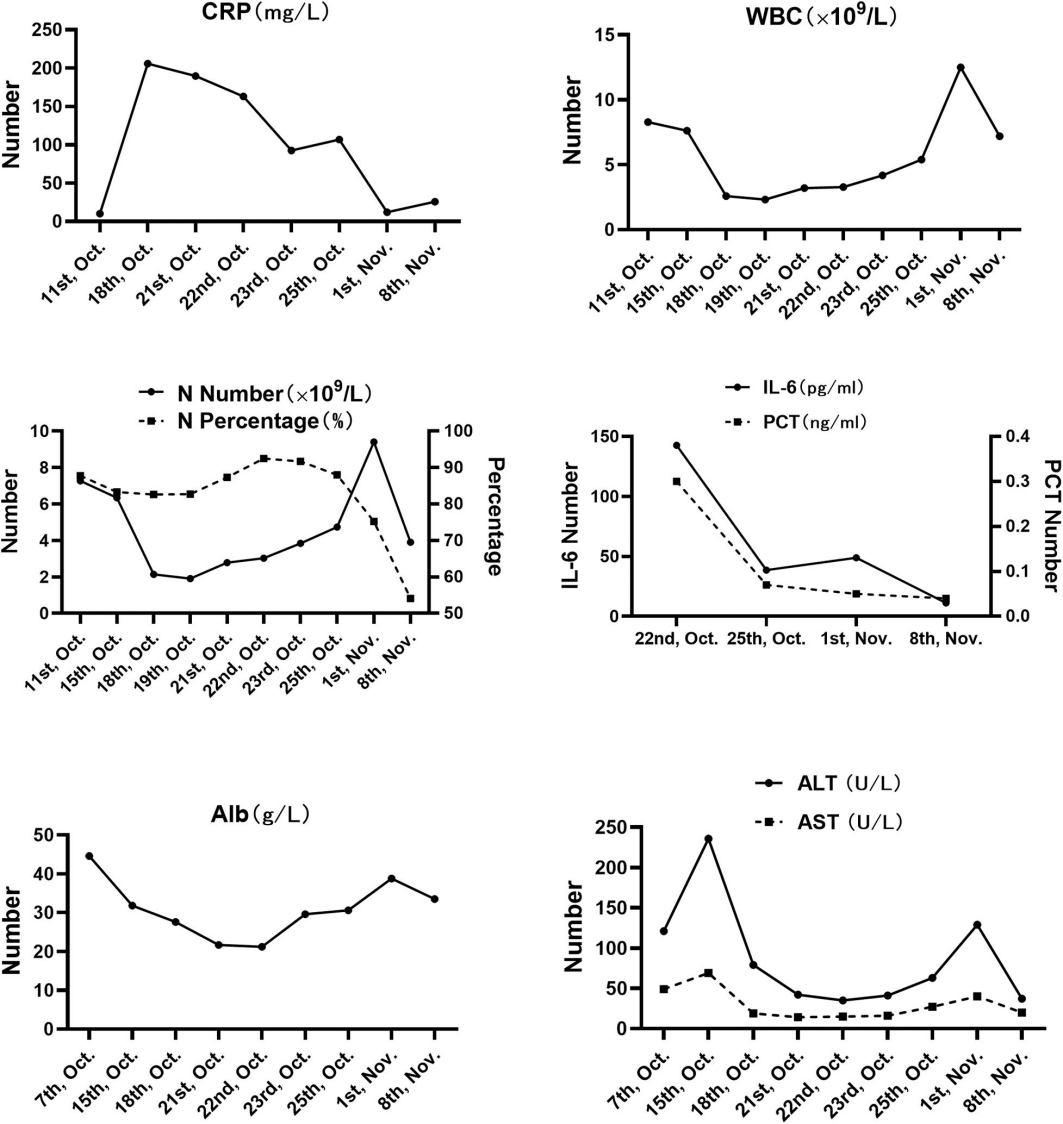
Trend chart of correlative laboratory indicators during the process of wound sepsis and TEN treatment. Infection indicators including CRP, WBC, NEUT, PCT and IL-6 and liver function indicators including AST and ALT increased significantly. Liver function indicators including Alb decreased significantly. When all the wounds were healed, the correlative indicators gradually returned to normal. TEN, toxic epidermal necrolysis; CRP, C-reactive protein; WBC, white blood cell; NEUT, neutrophils; PCT, procalcitonin; IL-6, interleukin-6; AST, aspartate aminotransferase; ALT, alanine aminotransferase; Alb, albumin.

Despite these interventions, his condition deteriorated on the fifth hospital day. He developed a high-grade fever (39.5 °C), tachycardia, and hypotension, accompanied by worsening skin detachment. Physical examination revealed a positive Nikolsky's sign, with epidermal loss involving approximately 92% of the total body surface area (TBSA) (Figures 1A–F). Mucosal involvement included oral, conjunctival, and genital erosions. Laboratory investigations showed leukocytosis (WBC 18.2 × 10⁹/L), elevated C-reactive protein (CRP 245 mg/L), procalcitonin rise (PCT 8.7 ng/mL), hypoalbuminemia (albumin 24 g/L), and mild transaminitis (ALT 68 U/L, AST 72 U/L) (Figure 2). Blood culture later grew multidrug-resistant Escherichia coli. Based on the above clinical manifestations, specialist physical examination findings, and laboratory test results, the clinical diagnosis of toxic epidermal necrolysis (TEN) complicated by wound sepsis was confirmed.

Given the extensive skin involvement and evolving sepsis, the patient was transferred to the burn intensive care unit (BICU) of our hospital on the 21st after onset. Management was guided by a multidisciplinary team (MDT) approach involving specialists from dermatology, critical care, infectious diseases, and wound care. The treatment strategy integrated major burn protocols: fluid resuscitation was initiated using the Parkland formula with hourly urine output monitoring; systemic corticosteroid dosage was gradually tapered under close monitoring; an air-fluidized bed was employed to minimize pressure and shear injury; wounds were cleansed with normal saline and dressed with nano-silver antimicrobial dressings combined with compound purple grass oil, changed every 48 h; targeted antibiotic therapy was adjusted per culture sensitivities; and nutritional support was intensified via enteral feeding with protein supplementation.

**Figure 3:**
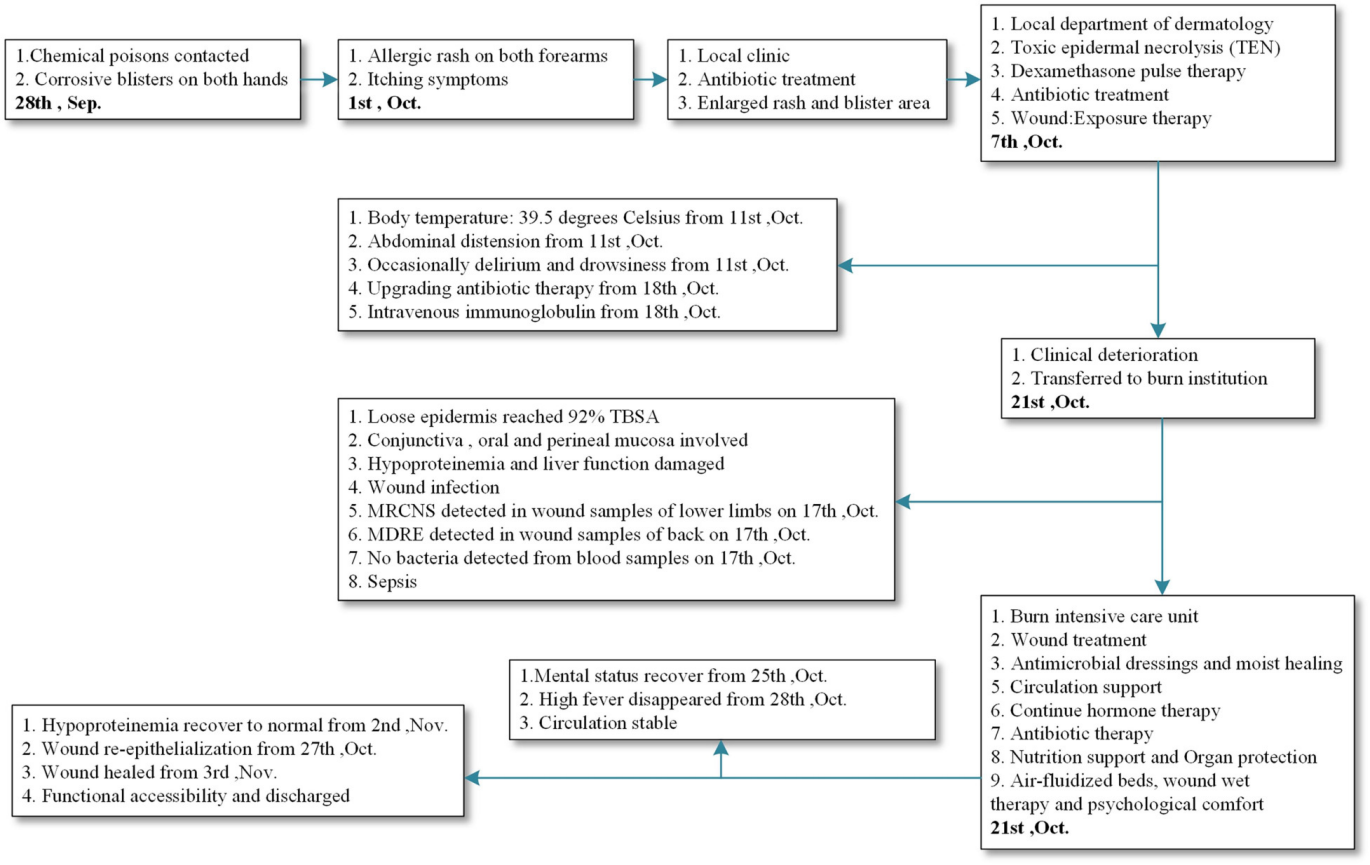
Timeline of the case, from dimethyl N-cyanodithioiminocarbonate contact to wound healing, including treatment for toxic epidermal necrolysis and wound sepsis. MRCNS, methicillin-resistant coagulase-negative staphylococci; MDRE, multidrug-resistant Escherichia coli; TBSA, total body surface area; TEN, toxic epidermal necrolysis; Sep., September; Oct., October; Nov., November.

Over the following two weeks, the patient's clinical status steadily improved. Fever subsided within seven days, and serial laboratory markers normalized (Figure 2). Re-epithelialization began on the 28th after onset, and complete wound closure was achieved on the 35th after onset without surgical intervention (Figures 1G–I). The patient was discharged home with fully healed skin, intact mucosal surfaces, and no functional impairment. A 12-month follow-up revealed that the patient achieved satisfactory recovery of global skin function without scar formation, pruritus, pigmentation, or associated functional impairments. There were no sequelae in the eyes, lips, and perineum, and no psychological disorders. A timeline summarizing the clinical course is provided in (Figure 3).

## 3 Discussion

Dimethyl N-cyanodithioiminocarbonate is a sulfur-containing organic compound with recognized industrial applications, yet its potential to induce severe dermatological emergencies such as toxic epidermal necrolysis (TEN) remains poorly documented. The compound is synthesized from highly toxic precursors including calcium cyanamide, carbon disulfide, and dimethyl sulfate, which may contribute to its potent sensitizing capacity (5). While the precise pathogenic mechanism remains to be fully elucidated, current evidence suggests that TEN triggered by such chemical exposures likely involves dysregulation of immune pathways such as the Fas-FasL system and a cytotoxic T-cell-mediated type IV hypersensitivity response, culminating in widespread keratinocyte apoptosis and epidermal detachment (6). Systemic complications—including hypoproteinemia, electrolyte disturbances, and secondary infections—are common and often life-threatening, particularly in cases with extensive skin involvement (2). In this case, the diagnosis of toxic epidermal necrolysis (TEN) was confirmed according to established criteria (7). Skin biopsy was not performed because of the extensive epidermal detachment and concerns about exacerbating wound sepsis and delaying wound healing. Key supporting factors included: diffuse epidermal necrosis with a positive Nikolsky's sign, oral, conjunctival, and genital mucosal involvement, and a clear temporal association with dimethyl N-cyanodithioiminocarbonate exposure (no alternative triggers identified). Differential diagnoses were excluded: Stevens-Johnson syndrome (SJS) (typically <10% TBSA, medication trigger), Staphylococcal scalded skin syndrome (SSSS) (no staphylococcal toxins, mucosal sparing), and Acute generalized exanthematous pustulosis (AGEP) (no pustules, discordant clinical/laboratory profile).

The present case is noteworthy as a rarely documented instance of dimethyl N-cyanodithioiminocarbonate-induced TEN managed with comprehensive burn care protocols. Based on age, heart rate, and epidermal damage area, our patient's SCORTEN score of 3 predicted a 35% mortality rate. While SCORTEN is the standard prognostic tool, a recent meta-analysis identifies non-SCORTEN predictors (such as sepsis) that increase the risk (8). Building upon anti-inflammatory and immunosuppressive therapy with methylprednisolone sodium succinate, we systematically implemented core burn-care strategies including precise fluid resuscitation (Third Military Medical University formula; 0.5–1.0 mL/kg/h urine output target), multi-organ support, rigorous infection control and specialized wound management (48 h nano-silver with compound purple grass oil dressing changes, air-fluidized bed pressure offloading), along with nutritional support (35–40 kcal/kg/d, 2.0–2.5 g/kg/d protein) (9). Early transfer to a burn intensive care unit (BICU) enabled a structured multidisciplinary approach that integrated these well-established burn-management principles—measures not routinely applied in conventional chemical-induced TEN treatment (10, 11). The use of an air-fluidized bed significantly reduced mechanical shear and pressure-related injury, while topical therapy with nano-silver dressings and compound purple grass oil supported a moist wound environment conducive to re-epithelialization (12, 13). This integrative protocol facilitated complete wound healing within two weeks without surgical intervention, underscoring the adaptability and efficacy of burn-derived strategies in chemical-induced TEN.

Notably, the clinical trajectory of dimethyl N-cyanodithioiminocarbonate-induced TEN exhibits distinct characteristics when compared with TEN caused by other industrial chemicals such as dimethyl sulfate (CAS number 77-78-1). While dimethyl sulfate is known for its potent systemic toxicity—often leading to rapid hepatorenal injury—dimethyl N-cyanodithioiminocarbonate appears to manifest primarily as an intense dermal sensitizer, with relatively milder initial systemic organ involvement (14). However, its capacity to provoke rapid and extensive epidermal necrolysis is striking; without timely intervention, localized erythema can progress to full-thickness epidermal detachment within days, as observed in our patient. This highlights the critical importance of early recognition and aggressive dermatological management, even when systemic parameters initially appear stable.

From an occupational health perspective, this case reinforces the urgent need for enhanced safety protocols in chemical manufacturing settings. Workers handling dimethyl N-cyanodithioiminocarbonate must be equipped with appropriate personal protective equipment and receive regular training on the compound's sensitizing potential. Furthermore, healthcare providers in both occupational and emergency medicine should be educated to recognize the early signs of chemical-induced TEN—initial localized rash should not be dismissed as trivial, as it may herald rapid systemic deterioration. Enterprises should establish clear exposure-response protocols, including immediate decontamination and urgent referral to specialized units capable of managing severe cutaneous adverse reactions.

Several limitations of this report should be acknowledged. As a single-case study, our findings cannot establish causality or define prognostic biomarkers. The absence of a control group precludes direct comparison with conventional TEN management strategies. Moreover, the unique pathophysiological and clinical profile of dimethyl N-cyanodithioiminocarbonate-induced TEN requires validation through larger, multi-center cohorts to better characterize its natural history, optimal treatment window, and long-term outcomes. Future studies should also explore potential serum biomarkers (e.g., sFasL) for early diagnosis and investigate targeted immunomodulatory therapies to further reduce morbidity.

In conclusion, the successful outcome in this high-mortality-risk case demonstrates that a burn-oriented, multidisciplinary care model can be effectively adapted to manage severe chemical-induced TEN. Early transfer to a specialized burn unit, combined with meticulous wound care and systemic support, may significantly improve survival and recovery. This report also serves as an alert to industrial and clinical communities regarding the potent sensitizing nature of dimethyl N-cyanodithioiminocarbonate and underscores the need for vigilant occupational safety measures and prompt medical intervention upon exposure.

## Data Availability

The original contributions presented in the study are included in the article/Supplementary Material, further inquiries can be directed to the corresponding author.
